# Unresectable Pancreatic Adenocarcinoma: Eight Years Later

**DOI:** 10.14740/wjon957w

**Published:** 2016-04-03

**Authors:** Kate Smiley, Reetu Malhotra, William Peche, John T. Langell

**Affiliations:** a Department of Surgery, University of Utah, 50 North Medical Drive, Salt Lake City, UT, USA; b Center for Medical Innovation, University of Utah, 10 North 1900 East, Eccles Library, Room 15, Salt Lake City, UT 84132, USA

**Keywords:** Pancreatic adenocarcinoma, Cancer, Unresectable cancer, Long-term survival

## Abstract

Pancreatic cancer is the fourth leading cause of cancer deaths in the United States, and is considered uniformly fatal when patients present with unresectable, advanced-stage disease at the time of diagnosis. Long-term survival of patients with advanced-stage pancreatic adenocarcinoma remains rare, despite advances in adjuvant chemoradiation protocols. A 73-year-old male presented to our emergency department with abdominal pain and a history of biopsy-proven, stage III pancreatic adenocarcinoma. His initial staging CT scan and trans-duodenal ultrasound had demonstrated a stage IIa (T3, N0, Mx) lesion. On surgical exploration, he was up-staged to stage III (T4, N0, Mx), noting encasement of the superior mesenteric vessels and involvement of the portal vein. He underwent palliative choledochojejunostomy and was treated with 4 months of oxaliplatin and capecitabine, with concurrent radiation therapy (50.4 Gy), followed by 4 months of gemcitabine. After 7 months, the patient withdrew from therapy due to treatment intolerance. He then turned to self-medication with non-traditional herbal therapies. After 3 years of surveillance, he was lost to follow-up until presenting to our facility with abdominal pain 8 years after his initial diagnosis. On diagnostic CT scan during his current presentation for abdominal pain, he was found to have no evidence of pancreatic cancer. Based on our review of the literature, we present the longest known survival of a patient with surgically unresectable pancreatic adenocarcinoma. Further study of this patient’s phenotypic or genotypic characteristics may provide insight into better therapeutic agents, or a predictive subset of patients who will benefit from specific chemotherapeutic options.

## Introduction

Pancreatic cancer is the fourth leading cause of cancer death in the United States, and portends a poor prognosis, in part due to delays in diagnosis caused by the subtleties in presentation and the limited efficacy of therapeutic options [1]. This is especially true with advanced-stage cancer. Currently, surgical resection is the only potentially curative therapeutic option for pancreatic cancer [2]. A recent case series including 269 patients, published in 2014, compared initially unresectable pancreatic cancer patients to those treated with palliative chemotherapy for advanced-stage recurrent disease. In this study, the 2-year survival rates were noted to be poor at 9.6% and 24.2%, respectively [3]. Prognosis for all patients diagnosed with pancreatic cancer is unfavorable, with overall 5-year survival rates less than 5% [4].

A PubMed query searching for long-term survival in patients with unresectable pancreatic cancer yields only two case reports. The first case report described a 68-year-old female with biopsy-proven, poorly differentiated adenocarcinoma with advanced-stage, surgically unresectable disease. The patient received gemcitabine therapy after palliative hepaticojejunostomy was reported to have attained a 4-year survival at the time of publication [5]. The second case report described a 61-year-old Japanese female with a 3.0 cm, stage III pancreatic adenocarcinoma invading the superior mesenteric vessels on CT imaging. At the time the publication was written, she was noted to have survived more than 65 weeks from presentation, after an initial palliative chemoradiation treatment regimen of gemcitabine 250 mg/m^2^/week for 6 weeks and radiation therapy of 50.2 Gy followed by 57 cycles of weekly gemcitabine [6].

Often, the only options for patients with unresectable disease are palliative surgical diversion and/or various adjuvant regimens consisting of gemcitabine in combination with a variety of other chemotherapy medications. Historically, 5-fluorouracil was the sole therapy for pancreatic adenocarcinoma; however, gemcitabine became the backbone of nearly all chemotherapy regimens in the mid-1990s [7]. In 1997, gemcitabine was shown to be superior to 5-fluorouracil in treatment of advanced, symptomatic pancreatic cancers, with a significant improvement in both pain and performance status, as well as increased overall survival [8]. In 2007, a hallmark multi-center randomized controlled trial established the superiority of the FOLFIRINOX regimen (oxaliplatin, irinotecan, fluorouracil, and leucovorin) versus gemcitabine, in terms of overall survival, for first-line treatment of metastatic pancreatic cancer [9].

## Case Report

**Figure 1:**
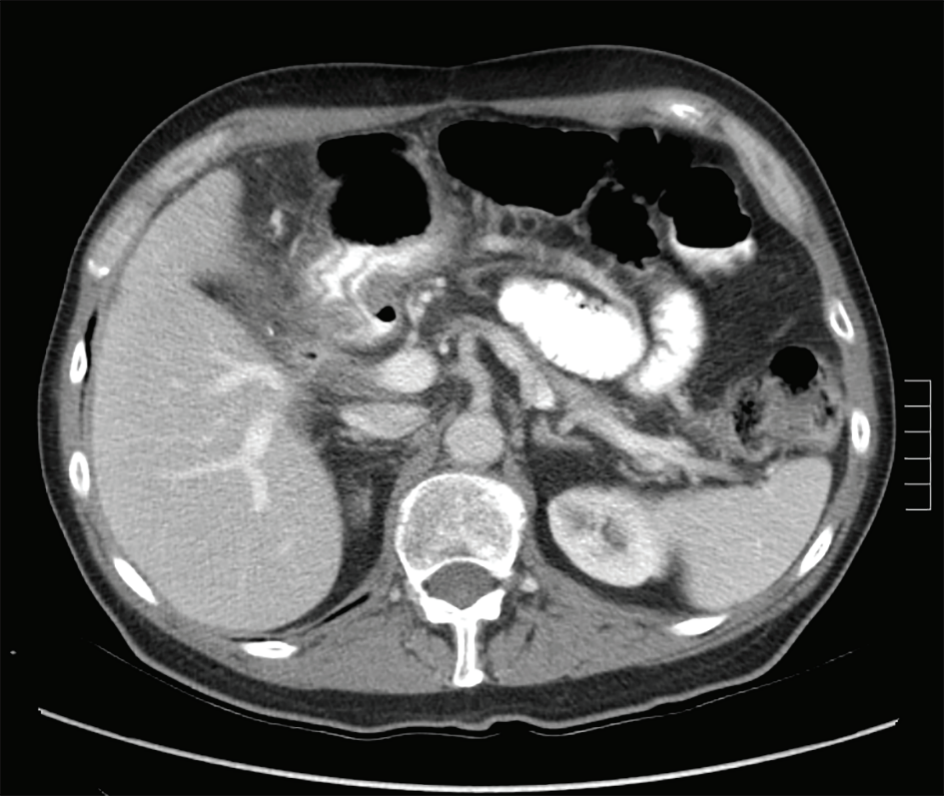
CT scan for evaluation of abdominal pain 8 years after diagnosis of pancreatic cancer demonstrating functional afferent limb syndrome.

A 73-year-old male with a history of palliative choledochojejunostomy for unresectable pancreatic adenocarcinoma 8 years prior presented to our emergency department with a 2-day history of vague abdominal pain, malaise and subjective fevers. Laboratory testing demonstrated an elevated white blood cell count of 13.9 × 10^3^/μL (reference range 3.7 - 8.4 × 10^3^/μL) and normal liver function tests and chemistry panel. As part of his diagnostic workup, a CT scan of the abdomen and pelvis was obtained, which demonstrated diffuse thickening of the hepaticobiliary limb concerning for a functional afferent limb syndrome due to bacterial overgrowth (Fig. 1). The patient was admitted to the surgical care unit and treated with a course of levaquin and flagyl. His symptoms and leukocytosis quickly resolved and he was discharged to home on hospital day 2.

**Figure 2:**
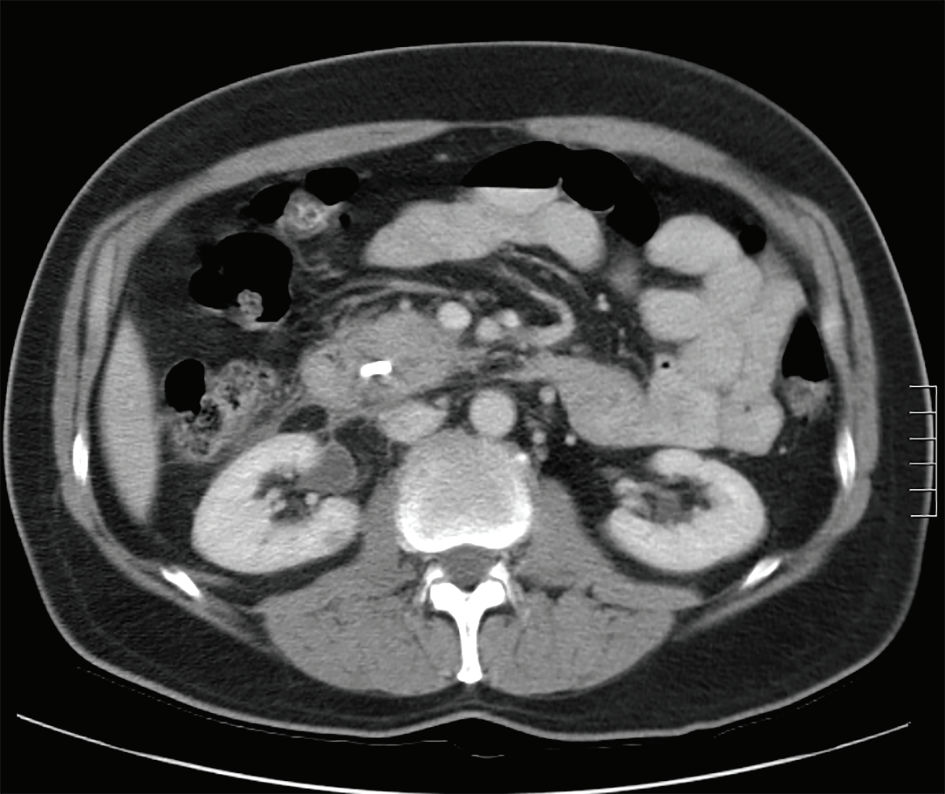
Initial CT scan demonstrating pancreatic head mass.

**Figure 3:**
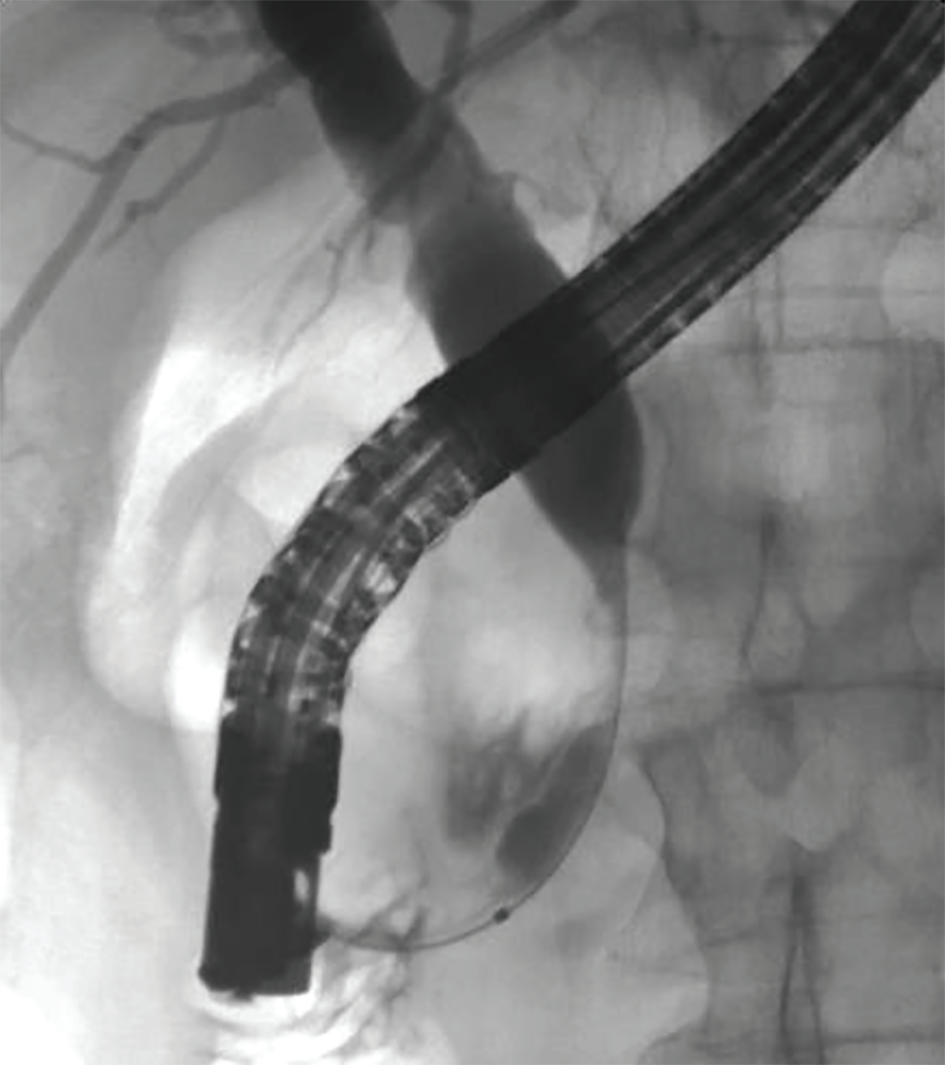
ERCP image of mass effect on the common bile duct.

**Figure 4:**
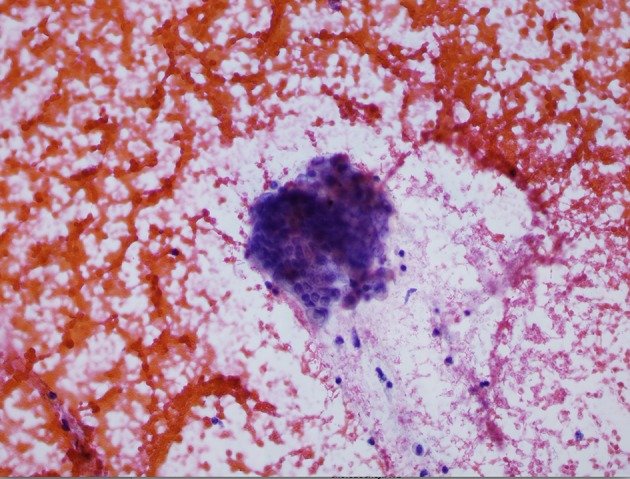
Pathology specimen from fine-needle aspiration.

A review of his medical records showed that he had presented to a community medical center 8 years prior with a 5-month history of gastroesophageal reflux, acholic stools, jaundice, dark urine and a total bilirubin of 6.0 mg/dL (reference range 0.2 - 1.4 mg/dL). His initial CT scan demonstrated a 4.6 cm mass in the head of the pancreas (Fig. 2). He was subsequently referred to our tertiary-care university medical center, where an ERCP and brushings demonstrated pancreatic ductal irregularities noted to be “suspicious for malignancy”, although pathological analysis was inconclusive (Fig. 3). His peak CA19-9 was 129 U/mL (reference range 0 - 37 U/mL) and his CEA was 1.7 ng/mL (reference range 0 - 5 ng/mL). An upper endoscopy with ultrasonography and fine-needle aspiration (FNA) was completed, demonstrating a 30 × 20 mm mass in the head of the pancreas with irregular borders, which obstructed the common bile duct and appeared to invade the portal vein. The endo-ultrasonographic appearance was consistent with adenocarcinoma, and he was staged as T3N0Mx. FNA specimens were independently reviewed by three cytopathologists from two institutions and were consistently read as well-differentiated mucinous adenocarcinoma (Fig. 4). The patient was otherwise in excellent health and deemed to be a good candidate for pancreaticoduodenectomy. On surgical exploration, the mass was found to be unresectable and the patient was upstaged to stage III, T4N0Mx. The operative report describes tumor completely encircling the superior mesenteric vessels, portal vein involvement and induration of the pancreas from head to tail. The surgical team aborted the planned pancreaticoduodenectomy and opted to perform a palliative cholecystectomy and choledochojejunostomy.

The patient was enrolled in a clinical trial at our affiliate cancer hospital, where he received 4 months of oxaliplatin and capecitabine, with concurrent 50.4 Gy radiation therapy, followed by 4 months of monotherapy with gemcitabine. This regimen is now considered first-line, although at the time it was the subject of a clinical trial. After nearly 7 months of adjuvant therapy, the patient developed treatment intolerance and associated complications including a duodenal ulcer and biliary enteric fistula. He declined further treatment. Repeat CT imaging demonstrated a decrease in the size of the pancreatic head mass from 4.6 to 2.9 cm; however, repeat endoscopic ultrasound again showed involvement of the portal and mesenteric vessels. The multidisciplinary cancer treatment planning committee deemed him inappropriate for surgical re-exploration.

**Figure 5:**
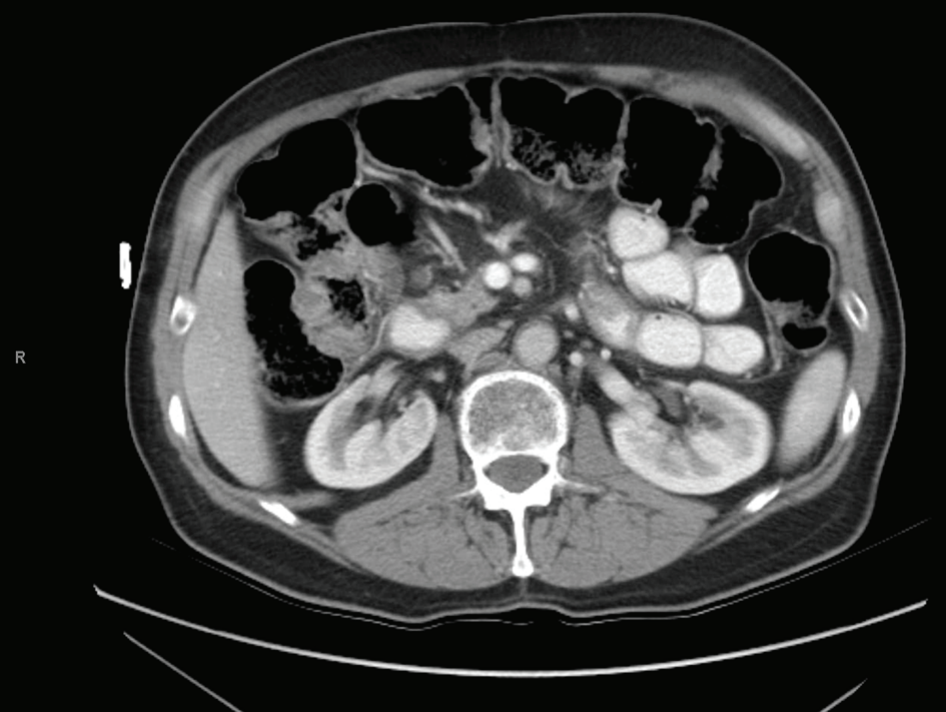
CT scan 3 years after aborted chemoradiation therapy.

The patient was followed for the next 3 years by the medical and radiation oncology services and subsequently ceased further surveillance. A chest radiograph and a CT of the abdomen and pelvis performed at his last oncology appointment 3 years post-operatively demonstrated no evidence of disease (Fig. 5). Since his final oncology visit, the patient had experienced no further abdominal pain and was reportedly in excellent health until his recent presentation to our emergency department.

The patient stated that after having developed treatment intolerance and being informed that he was not a candidate for surgical resection, he explored non-traditional therapies. Shortly after withdrawing from the chemotherapy trial, he began long-term daily consumption of two nutraceutical supplements: Protandim and Essiac Tea.

## Discussion

Pancreatic cancer continues to be one of the most challenging areas in oncology, with an exceptionally poor prognosis for the majority of patients. Longevity with advanced-stage disease is quite limited and there are very few cases of long-term survival after palliative surgery and chemoradiation in the literature. Chemotherapy has demonstrated limited success for advanced pancreatic cancers, and more efficacious therapies are needed.

Based on our review of the literature, this is the first presented case of a patient with surgically unresectable disease who had a complete response to adjuvant chemoradiation therapy for pancreatic cancer. Given this patient’s oncologic results and long-term survival, we were skeptical of his initial diagnosis. We therefore obtained his original pathology slides and had them read by three pathologists from two institutions who were blinded to the patient’s diagnosis and previous pathologic interpretations.

Patients with advanced pancreatic cancer sometimes seek alternative remedies when conventional therapies either fail or are deemed futile. In this case, our patient chose to use Protandim and Essiac Tea. Promoters assert that these agents serve as immunomodulators, analgesics, appetite stimulants and antineoplastic agents [10, 11]. There is however no valid scientific evidence to support these assertions. To the contrary, an article published in Nutrition and Cancer recently compared Essiac Tea to paclitaxel to study its anti-proliferative effects in prostate cancer cells, both *in vivo* and *in vitro*. The study demonstrated no significant difference in tumor size, cell cycle distribution effects, or cell toxicity after treatment with Essiac Tea versus control [12]. Although we cannot completely discount the potential impact of its use, either alone or in combination with standard chemotherapeutic regimens, caution should be exercised in drawing conclusions about its efficacy in this case study. The anti-proliferative effects of Essiac Tea and Protandim have not been studied in pancreatic cancer. Based on the observation from this case report, it might be interesting to conduct a clinical trial of standard chemotherapy followed by long-term application of the two nutraceutical supplements taken by this patient: Protandim and Essiac Tea.

### Conclusion

Based on a review of the literature, we present the longest known survival of a patient with surgically unresectable, biopsy-proven pancreatic adenocarcinoma. This patient has survived 8 years since his initial diagnosis with no active disease noted on imaging studies. Further scientific review of this index case may provide insight into better therapeutic options or provide for a predictive subset of patients who will benefit from specific chemotherapeutic options.
